# Characterization and mechanism of aflatoxin degradation by a novel strain of *Trichoderma reesei* CGMCC3.5218

**DOI:** 10.3389/fmicb.2022.1003039

**Published:** 2022-10-13

**Authors:** Xiaofeng Yue, Xianfeng Ren, Jiayun Fu, Na Wei, Claudio Altomare, Miriam Haidukowski, Antonio F. Logrieco, Qi Zhang, Peiwu Li

**Affiliations:** ^1^Oil Crops Research Institute of the Chinese Academy of Agricultural Sciences, Wuhan, China; ^2^Key Laboratory of Biology and Genetic Improvement of Oil Crops, Ministry of Agriculture and Rural Affairs, Wuhan, China; ^3^Institute of Quality Standard and Testing Technology for Agro-products, Shandong Academy of Agricultural Sciences, Jinan, China; ^4^Institutions of Agricultural Product Quality Standard and Testing Research, Tibet Academy of Agricultural and Animal Husbandry Sciences, Lhasa, China; ^5^Institute of Sciences of Food Production, National Research Council, Bari, Italy; ^6^Hubei Hongshan Lab, Wuhan, China

**Keywords:** aflatoxins, aflatoxin B_1_, *Aspergillus flavus*, *Trichoderma reesei* CGMCC3.5218, degradation, detoxification

## Abstract

Aflatoxins, which are produced mainly by *Aspergillus flavus* and *A. parasiticus*, are recognized as the most toxic mycotoxins, which are strongly carcinogenic and pose a serious threat to human and animal health. Therefore, strategies to degrade or eliminate aflatoxins in agro-products are urgently needed. We investigated 65 *Trichoderma* isolates belonging to 23 species for their aflatoxin B_1_ (AFB_1_)-degrading capabilities. *Trichoderma reesei* CGMCC3.5218 had the best performance, and degraded 100% of 50 ng/kg AFB_1_ within 3 days and 87.6% of 10 μg/kg AFB_1_ within 5 days in a liquid-medium system. CGMCC3.5218 degraded more than 85.0% of total aflatoxins (aflatoxin B_1_, B_2_, G_1_, and G_2_) at 108.2–2323.5 ng/kg in artificially and naturally contaminated peanut, maize, and feed within 7 days. Box–Behnken design and response surface methodology showed that the optimal degradation conditions for CGMCC3.5218 were pH 6.7 and 31.3°C for 5.1 days in liquid medium. Possible functional detoxification components were analyzed, indicating that the culture supernatant of CGMCC3.5218 could efficiently degrade AFB_1_ (500 ng/kg) with a ratio of 91.8%, compared with 19.5 and 8.9% by intracellular components and mycelial adsorption, respectively. The aflatoxin-degrading activity of the fermentation supernatant was sensitive to proteinase K and proteinase K plus sodium dodecyl sulfonate, but was stable at high temperatures, suggesting that thermostable enzymes or proteins in the fermentation supernatant played a major role in AFB_1_ degradation. Furthermore, toxicological experiments by a micronucleus assay in mouse bone marrow erythrocytes and by intraperitoneal injection and skin irritation tests in mice proved that the degradation products by CGMCC3.5218 were nontoxic. To the best of our knowledge, this is the first comprehensive study on *Trichoderma* aflatoxin detoxification, and the candidate strain *T. reesei* CGMCC3.5218 has high efficient and environment-friendly characteristics, and qualifies as a potential biological detoxifier for application in aflatoxin removal from contaminated feeds.

**GRAPHICAL ABSTRACT:**
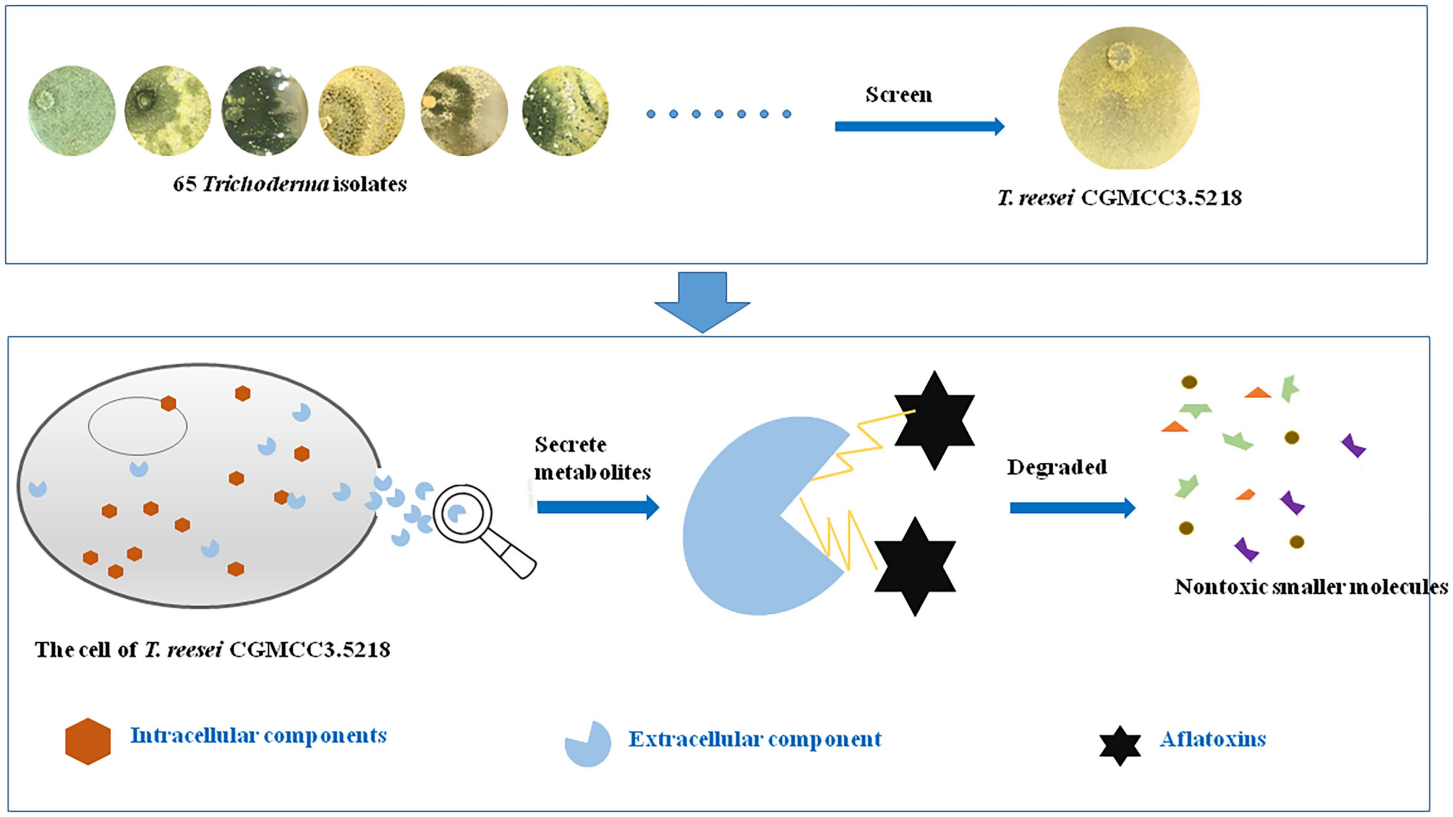


## Introduction

Aflatoxins were recognized as a group of small molecules containing difuran rings and lactone rings. The aflatoxins occurring in agricultural products are categorized as B_1_, B_2_, G_1_, and G_2_ (toxicity grade: AFB_1_ > AFG_1_ > AFB_2_ > AFG_2_) (Marin et al., 2013; Heshmati et al., 2021). Aflatoxin B_1_ (AFB_1_), the most hepatotoxic, mutagenic, and carcinogenic member of the aflatoxin family, has been classified as a Group I carcinogen by the International Agency for Research on Cancer (IARC, 2002). However, agricultural products such as peanuts, maize, wheat, rice and spices are suffering from a high risk of aflatoxin contamination in many countries (Pitt and Hocking, 2006; Keller et al., 2013; Nejad et al., 2014; Eslami et al., 2015). The ingestion of such aflatoxin-contaminated feeds can lead to slow growth, illness, and even death (Bennett and Klich, 2003). Aflatoxins can be biotransformed into metabolites and toxic residues that contaminate meat, eggs, and milk (Nejad et al., 2019; Heshmati et al., 2020). For example, AFB_1_ and AFB_2_ can be biotransformed into aflatoxins M_1_ and M_2_, respectively, by hydroxylation in animal livers, and subsequently excreted in the urine and milk, posing a serious threat to the safety of dairy foods (Battacone et al., 2005; Kamkar et al., 2014; Nejad et al., 2020).

Many studies have been carried out to explore an effective strategy to degrade aflatoxins to a tolerable level. Although several physical approaches, including γ-radiation treatment and microwave heating (Pankaj et al., 2018), and chemical reactions, e.g., oxidation by ozone (Luo et al., 2013), have achieved some success, these approaches have limitations, such as residual toxicity, losses in nutritional value, and the modification of food or feed properties. The biological degradation of aflatoxins, which mainly includes biodegradation by bacteria, fungi, and yeasts or by their enzymes (Guo et al., 2020; Loi et al., 2020), has been proved to be the most effective, specific, and eco-friendly solution to degrade aflatoxins to nontoxic or less toxic compounds in foods and feeds (Ji et al., 2016).

The fungal genus *Trichoderma* includes more than 100 species commonly found in soil and root ecosystems, and occasionally in grains and rotten woods. Members of this genus have been well documented to have high value as biocontrol agents, whose mode of action includes the production of secondary metabolites with antibiotic activity (Vinale et al., 2006; Ren et al., 2020). More than 1,000 secondary metabolites have been identified from *Trichoderma* spp. over the years (Hermosa et al., 2014), some of which have been utilized for biocontrol of plant diseases (Calistru et al., 1997; Braun et al., 2018) or for breaking down complex molecules, such as lignin and cellulose within industrial processes (Juhász et al., 2005; Gusakov, 2011). Although several *Trichoderma* species, including *T. harzianum*, *T. reesei*, *T. koningii*, and *T. asperellum*, have been demonstrated to have the ability to degrade hydrocarbon compounds, such as polycyclic aromatic hydrocarbons (PAHs), crude oil, and resins (Zafra and Cortés-Espinosa, 2015), reports on the biodegradation of small molecule contaminants, particularly on degradation of aflatoxins, by *Trichoderma* spp. are still sparse (Palonen et al., 2004; Pribowo et al., 2012; Hackbart et al., 2014).

Gene sequencing and the analysis of biosynthetic pathways for secondary metabolites have provided insight into the extraordinary capacity of *T. reesei* to metabolize and degrade small molecular contaminants (Martinez et al., 2008; Zeilinger et al., 2016). The objectives of the present study were to evaluate the AFB_1_-degradation efficiency of various *Trichoderma* isolates and to evaluate the *T. reesei* strains with the strongest AFB_1_ degradation capability. Furthermore, the mechanism of AFB_1_ degradation by *T. reesei* was revealed, which provided a theoretical basis for the degradation of AFB_1_ in agricultural products by enzymes, and then promote the rapid development of aflatoxin detoxification by the green, efficient and universal enzymes.

## Materials and methods

### *Trichoderma* strains, chemicals, and media

A total of 65 *Trichoderma* isolates from different geographical origins, including China, Italy, the United States, Australia and Borneo, and different sources such as soil, fruit and wood were examined (Supplementary Table S1). In contrast, the sequences of the internal transcribed spacer regions ITS-1 and ITS-2 of the nuclear rDNA are relatively stable, which represents a moderate amount of information and could be used to identify the isolates (Fanelli et al., 2018; Ren et al., 2022). All the isolates were molecularly characterized by analysis of the sequences of the internal transcribed spacer regions ITS-1 and ITS-2 of the nuclear rDNA. The standards of aflatoxins were purchased from Sigma-Aldrich (St. Louis, MO, United States). Water was purified by a Milli-Q purification system (Millipore, Bedford, MA, United States). All other chemicals and reagents were analytically pure or better and purchased from local chemical stores. Yeast extract peptone dextrose (YPD: 5 g yeast extracts, 10 g peptone, 10 g dextrose, and 1 L H_2_O, pH 7.2) medium was used for the liquid culture of *Trichoderma*. Potato dextrose agar (PDA) was used for the solid culture of *Trichoderma* and *A. flavus*.

### Determination the potential *Trichoderma* isolates capable of detoxifying AFB_1_

The assay was carried out as described by Hackbart et al. (2014) with some modification. The standard of AFB_1_ was diluted in YPD medium to a final concentration of 50 ng/kg, and then each well of a 12-well plate (Costar Inc., Cambridge, MA, United States) was filled with 2 ml of the medium and inoculated with one 6-mm-diameter mycelium plug that was excised with a cork-borer from a 5-day-old culture of *Trichoderma* on PDA. In parallel, controls were made with YPD medium supplemented with the same amount of AFB_1_ but without inoculation with *Trichoderma*. After 1, 3, and 7 days of incubation at 28°C under static conditions in the dark, 1 ml of the supernatant was collected from each one of triplicated wells, filtered through a 0.22-μm-filter membrane, and analyzed by HPLC-FLD (high-performance liquid chromatography with fluorescence detector) for determination of AFB_1_. The AFB_1_ removal ratio was calculated using the following formula (F_1_): F_1_ (%) = (C_1_ - S_1_) / C_1_ × 100, where S_1_ was the AFB_1_ concentration in wells supplemented with AFB_1_ and inoculated with *Trichoderma*, and C_1_ was the AFB_1_ concentration in the control wells.

### Effects of various conditions on AFB_1_ removal ability by *Trichoderma reesei* CGMCC3.5218

According to the results of the experiment described above, *T. reesei* CGMCC3.5218 had a great performance in AFB_1_ removal and was thus used for further study. The effects of incubation time, culture temperature, pH, nutritional sources, and metal ions on AFB_1_ removal were evaluated as described by Zhang et al. (2014) and Xu et al. (2016). The assay was carried out as follows: tested incubation time with 1, 3, 5, 7, and 10 days; tested pH values with 2.2, 4.5, 5.4, 6.5, 7.2, 8.3, and 9.5, prepared by adjusting the pH of YPD (7.2) with 12 M HCl or 2 M NaOH; tested incubation temperatures with 4, 20, 28, 37, 45, and 50°C; the effects of six growing media were compared: Czapek-Dox broth (CDB, 0.1% NaNO_3_, 0.1% K_2_HPO_4_, 0.05% MgSO_4 ▪_ 7H_2_O, 0.05% KCl, 0.001% FeSO_4_, and 2% sucrose), malt extract broth (MEB, 1.7% malt extract, and 0.3% peptone), nutrient broth (NB, 1% peptone, 0.3% beef extract, and 0.5% NaCl), potato dextrose broth (PDB), liquid Sabouraud broth (LSB, 1% peptone, and 4% dextrose), and YPD; tested metal ions Al^3+^, Ca^2+^, Cu^2+^, Fe^2+^, K^+^, Mg^2+^, Mn^2+^, Na^+^, and Zn^2+^ from AlCl_3_, CaCl_2_, CuSO_4_, FeSO_4_, KCl, MgCl_2_, MnSO_4_, NaCl, and ZnSO_4_, respectively, all at the concentration of 10 mmol/l (Mwakinyali et al., 2019). The liquid media were prepared as described and then supplemented with AFB_1_ standard to a final concentration of 500 ng/kg. Two milliliter of each medium were transferred into each well of the 12-well plate, inoculated with one plug of *T. reesei* CGMCC3.5218. The plates used for testing incubation time were incubated at 28°C for 1, 3, 5, 7, or 10 days. The plates used for testing culture temperatures were incubated at 4, 20, 28, 37, 45, or 50°C for 3 days. The plates used for other tests were incubated at 28°C for 3 days. Controls were prepared in the same way, except that controls were not inoculated with *Trichoderma*. After different days of incubation, the supernatants were collected, filtered, and analyzed for AFB_1_ detection. The degradation ratio was calculated with the following formula (F_2_): F_2_ (%) = (C_2_–S_2_)/C_2_ × 100, where S_2_ was the AFB_1_ concentration in testing wells, and C_2_ was the AFB_1_ concentration in control wells.

### Box–Behnken design

To investigate cultivation methods that can be used to obtain the optimal efficiency of AFB_1_ degradation, we used Box–Behnken (BB) design and response surface methodology (RSM) to optimize the values and interaction effects of the factors that affected the AFB_1_ removal (Mugwagwa and Chimphango, 2019). The incubation time, culture temperature, and pH were identified as three effective factors (*X*_1_, *X_2_*, and *X_3_*, respectively) and were investigated at 3 levels (−1, 0, and 1) (Supplementary Table S2) (Wang et al., 2015b). The experimental design matrix and response values are shown in Supplementary Table S3.

### Pattern analysis of AFB_1_ removal by *Trichoderma reesei* CGMCC3.5218

To determine functional detoxification component, we analyzed the effects of the culture supernatant, intracellular components, and mycelium of CGMCC3.5218 on the AFB_1_ removal ability. The assay was carried out as described in Li et al. (2018) with some modifications. To confirm that the removal of AFB_1_ did not occur because of the adsorption by the cell wall, a desorption experiment was conducted. AFB_1_ was supplemented in YPD medium (100 ml) in a flask to a final concentration of 500 ng/kg. Then, five 6-mm-diameter CGMCC3.5218 plugs were added to the flask and incubated for 7 days with constant shaking (125 rpm) at 28°C. The supernatants were collected for AFB_1_ analysis by HPLC-FLD. The remaining mycelium pellets were also collected, gently rinsed twice with phosphate-buffered saline (PBS, 10 mM sodium phosphate buffer containing 137 mM NaCl and 2.68 mM KCl, pH 7.4) to wash off the medium, and then treated with 25 ml of PBS, methanol, or acetonitrile with constant shaking (200 rpm) for 1 h to elute the AFB_1_ adsorbed by the pellets. Afterward, the pellets were discarded and the solvents were recovered for AFB_1_ determination by HPLC-FLD.

### Degradation dynamics of AFB_1_ by *Trichoderma reesei* CGMCC3.5218 culture supernatant

The time-course of AFB_1_ degradation by the culture supernatant was studied. The supernatant was supplemented with AFB_1_ standard to 50, 100, 400, 800, and 1,500 ng/kg and incubated at 37°C under static conditions in the dark. Triplicated samples were collected separately after 0, 12, 24, 48, 72, and 96 h (Mwakinyali et al., 2019). The initial pH of the supernatant was approximately 7 and was then adjusted to different values of pH, *viz.* 3, 4, 5, 6, 7, 8, and 9. Three milliliter aliquots of the supernatant at different pH values were transferred into new tubes and supplemented with AFB_1_ standard to the final concentration of 500 ng/kg. Samples were collected after incubated at 37°C in the dark for 24 h. Same aliquots of PBS buffer treated in the same manner of samples were used as a control. The degradation rate of AFB_1_ was calculated using formula (F_3_): F_3_(%) = (C_3_–S_3_)/C_3_ × 100, where C_3_ refers to the concentration of AFB_1_ in the control group, and S_3_ refers to the concentration of AFB_1_ in the sample group.

### Effects of heat, proteinase K, sodium dodecyl sulfonate, and ethylene diamine tetraacetic acid on the activity of culture supernatant to degrade AFB_1_

To investigate whether AFB_1_ degradation was caused by enzymatic digestion, the CGMCC3.5218 supernatant was subjected to the following treatments: heating at 121°C for 30 min; separate treatment with 1 mg/ml proteinase K and 1 mg/ml proteinase K plus 1% (w/v) sodium dodecyl sulfonate (SDS) at 55°C for 1 h; treatment with 10 mmol/l EDTA at 37°C for 2 h (Sangare et al., 2014; Wang et al., 2017). PBS buffer was used for the controls and treated in the same way. After the treatments, samples and controls were supplemented with standard AFB_1_ to the final concentration of 500 ng/kg, kept at 37°C for 24 h under static conditions in the dark, and then analyzed by HPLC-FLD to determine the AFB_1_ content.

### Aflatoxin degradation by *Trichoderma reesei* CGMCC3.5218 in peanut cake, maize, and completed feed

The blank peanut cakes, maize kernels, and animal feed were purchased from the local market. Naturally contaminated maize samples were collected from Shandong Province, China, and stored in our laboratory. The blank samples were inoculated with conidial suspensions of the aflatoxigenic *A. flavus* strain 3.4408 to produce artificially contaminated samples (Branà et al., 2017). The contaminated samples were autoclaved (121°C for 30 min) and then finely ground into powder. The powder (0.6 g) were transferred into test tubes filled with 20 ml of melted agar (15 g/l) supplemented with 1.0 g/l K_2_HPO_4_, 0.5 g/l MgSO_4▪_7H_2_O, 0.5 g/l CaCl_2_, and 0.5 g/l NaCl. After autoclaving (121°C for 15 min), the mixture was thoroughly mixed by a vortex mixer and poured into 9-cm-diameter Petri dishes. Then, every dish was inoculated with three 6-mm-diameter plugs of CGMCC 3.5218. After 1, 3, and 7 days of incubation at 28°C, the samples (approximately 5 g) were excised along the radius of the colony, precisely weighed, transferred to test tubes, and then extracted with 25 ml methanol–water (80:20, v/v) solution. The samples were then vortexed, centrifuged, filtered and analyzed by HPLC-FLD for the determination of aflatoxins B_1_, B_2_, G_1_, and G_2_.

### Toxicity analysis of AFB_1_ degradation products

The culture supernatant containing AFB_1_ degradation products were prepared. The toxicity of the supernatant was evaluated by a micronucleus assay in mouse bone marrow erythrocytes (an acute oral toxicity test) and by intraperitoneal injection and skin irritation tests in mice (half male and half female). All of the tests were carried out in accordance with China National Standards GBZ/T 240.11–2011 and China Agriculture Industry Standard NY/T 1109–2017 by the Guangdong Detection Center of Microbiology, which is a Chinese authoritative organization providing toxicity analysis services.

### Aflatoxin determination by HPLC-FLD

The aflatoxin determination was carried out according to Ren et al. (2022). Aflatoxins were analyzed by an HPLC-FLD apparatus of the series 1,100 from Agilent Technologies (Santa Clara, CA, United States), with a fluorescence detector and an HPLC C-18 column of 4.6-mm inner diameter, 250-mm length, and 5-μm particle size. The mobile phase was methanol–water (45: 55, v/v) with a 0.8 ml min^−1^ flow rate and 20 min of total run time. The injection volume was 10 μl, and the excitation and emission wavelengths were 360 and 440 nm, respectively.

### Statistical analysis

Data were analyzed by the softwares Origin 9.0 (Origin Lab Corp., Northampton, MA, United States) and GraphPad Instat 3.0 (GraphPad Software, San Diego, CA, United States). One-way analysis of variance (ANOVA) and the Tukey–Kramer test for multiple comparisons were used to analyze the data. Differences were considered extremely significant when *p* < 0.001, significantly significant when *p* < 0.01, significant when *p* < 0. 05 and have no differences when *p* > 0.05.

## Results and discussions

### Screening and characterization of the most promising *Trichoderma* isolates for aflatoxin detoxification

**Figure 1:**
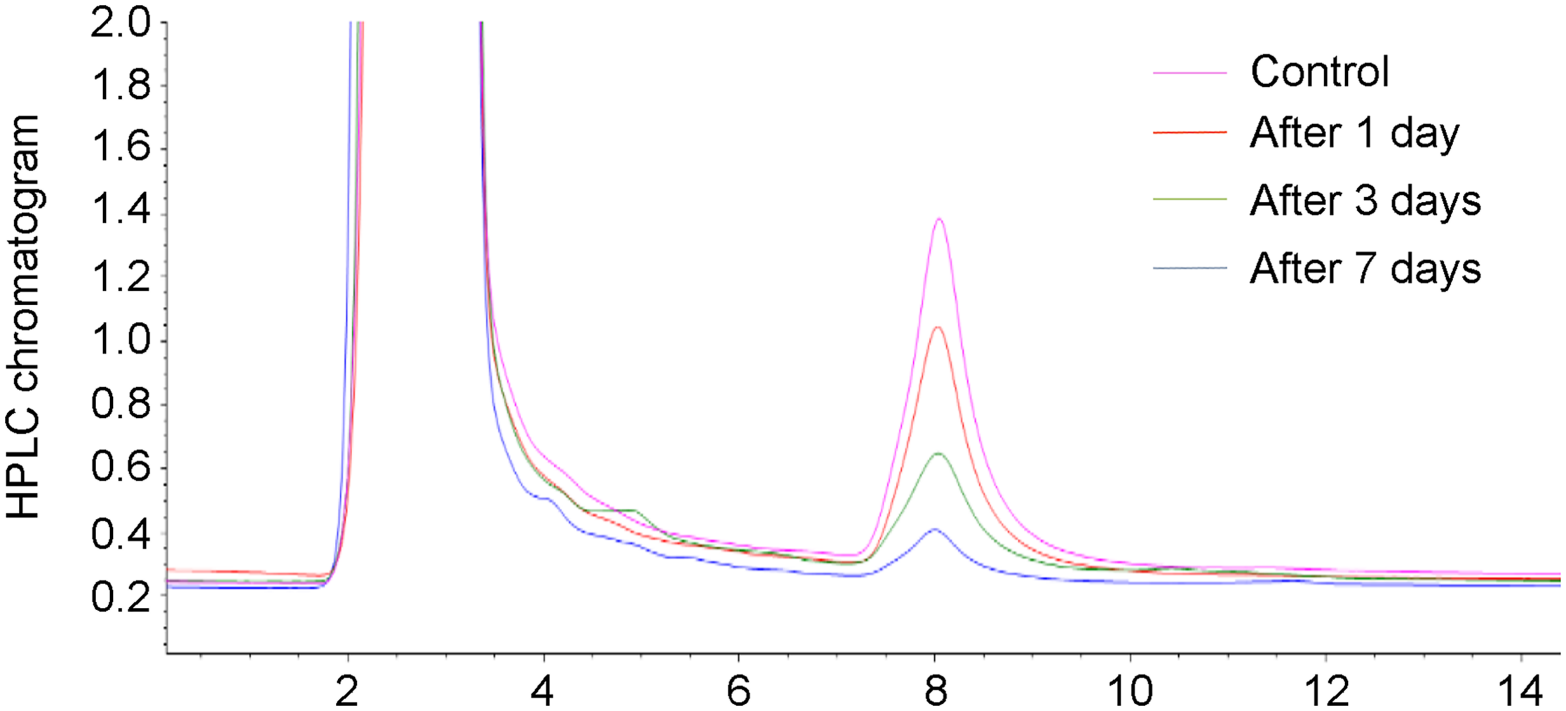
Chromatograms relevant to the aflatoxin B_1_ (AFB_1_) (50 ng/kg) in the control and YPD liquid medium after 1, 3, and 7 days of removal by a *Trichoderma* isolate.

In this study, the HPLC chromatograms showed that the peak areas for AFB_1_ concentration clearly decreased with the increase of incubation time, indicating the effective removal of AFB_1_ by *Trichoderma* (Figure 1). It is noteworthy that we tested the AFB_1_ removal ratios of the 65 collected *Trichoderma* isolates to determine the AFB_1_ removal abilities of different *Trichoderma* isolates. We found that the 65 *Trichoderma* isolates possessed different capacity to remove AFB_1_. After 7 days of incubation, 49 of the 65 isolates could remove more than 70% of AFB_1_. For species, including *T. citrinoviride*, *T. erinaceum*, *T. harzianum*, *T. koningii*, *T. koningiopsis*, and *T. viride*, the results of one-way ANOVA showed that different strains within the same species had significantly different AFB_1_ removal capacities, indicating intraspecific variability (Supplementary Table S1).

**Figure 2:**
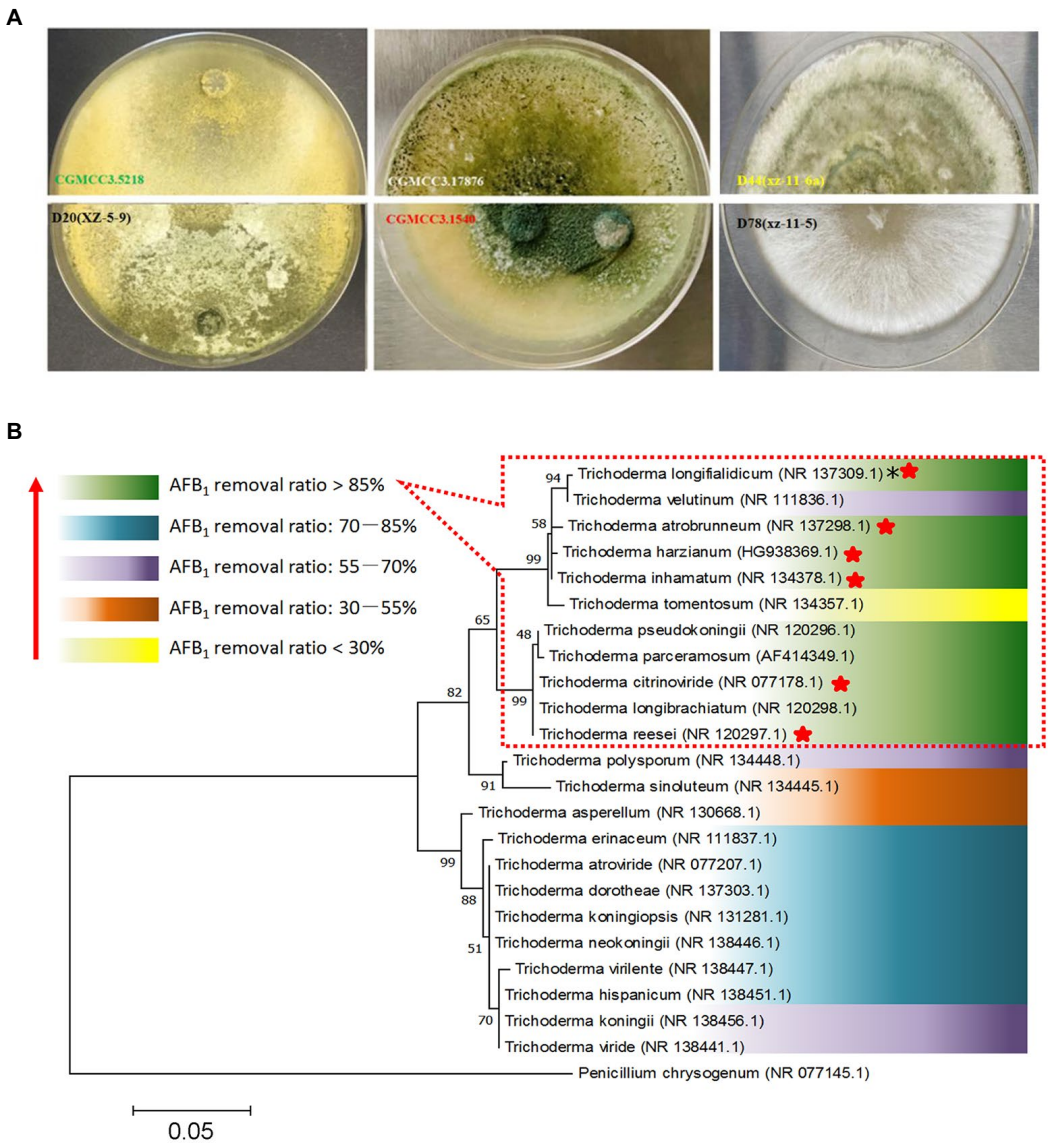
Screening of different *Trichoderma* isolates for aflatoxin detoxification. **(A)** The colonial morphology of *T. reesei* CGMCC3.5218, *T. citrinoviride* D20 (xz-5-9), *T. harzianum* CGMCC3.17876, *T. atrobrunneum* CGMCC3.1540, *T. inhamatum* D44 (Xz-11-6a), and *T. longifialidicum* D78 (xz-11-5) cultured in potato dextrose agar (PDA) after 5 days of incubation at 28°C in the dark. **(B)** The relationships between *Trichoderma* evolution and aflatoxin B_1_ (AFB_1_) removal ability. The abilities of different species to remove AFB_1_ are divided into five levels, which are represented by different colors according to the mean value of AFB_1_ removal ratios of different intra-species strains. *NR: The code of the reference internal transcribed spacer (ITS) sequence obtained from the website of the National Center for Biotechnology Information (NCBI). The top six species having a higher AFB_1_ (50 ng/kg) removal efficiency were marked by red stars.

The principles for defining the more efficient *Trichoderma* isolates were AFB_1_ removal ratio higher than 80.0% after 3 days of incubation and 100.0% after 7 days of incubation. According to the data in Supplementary Table S1, isolates *T. atrobrunneum* CGMCC3.1540, *T. citrinoviride* D20 (xz-5-9), *T. harzianum* CGMCC3.17876, *T. inhamatum* D44 (Xz-11-6a), *T. longifialidicum* D78 (xz-11-5), and *T. reesei* CGMCC3.5218 met these principles and were considered the top six isolates with high AFB_1_ removal efficiency. The morphology of colonies of the six strains are shown in Figure 2A. The morphology and visual observations during the experimental process showed that the six strains had similar growth rates, maturity periods, and colony sizes, whereas the colony colors, mycelial structures, and spore morphology were somewhere different (Figure 2A). To analyze the genetic relationships between these species with a higher AFB_1_ removal efficiency, a phylogenetic tree was first built using MEGA 7.0 software with *Penicillium chrysogenum* as the root, according to internal transcribed spacer (ITS) sequences of the species. The parameters of the phylogenetic tree are shown in Supplementary Table S4. Phylogenetic analysis indicated that the *Trichoderma* isolates with similar AFB_1_ removal abilities were genetically the closest to each other. For example, the top six isolates, which had AFB_1_ removal ratios higher than 85%, that belong to the species *T. atrobrunneum*, *T. citrinoviride*, *T. harzianum*, *T. inhamatum*, *T. longifialidicum*, and *T. reesei,* respectively, shared approximately 90% ITS sequence identity with each other (Figure 2B).

**Figure 3:**
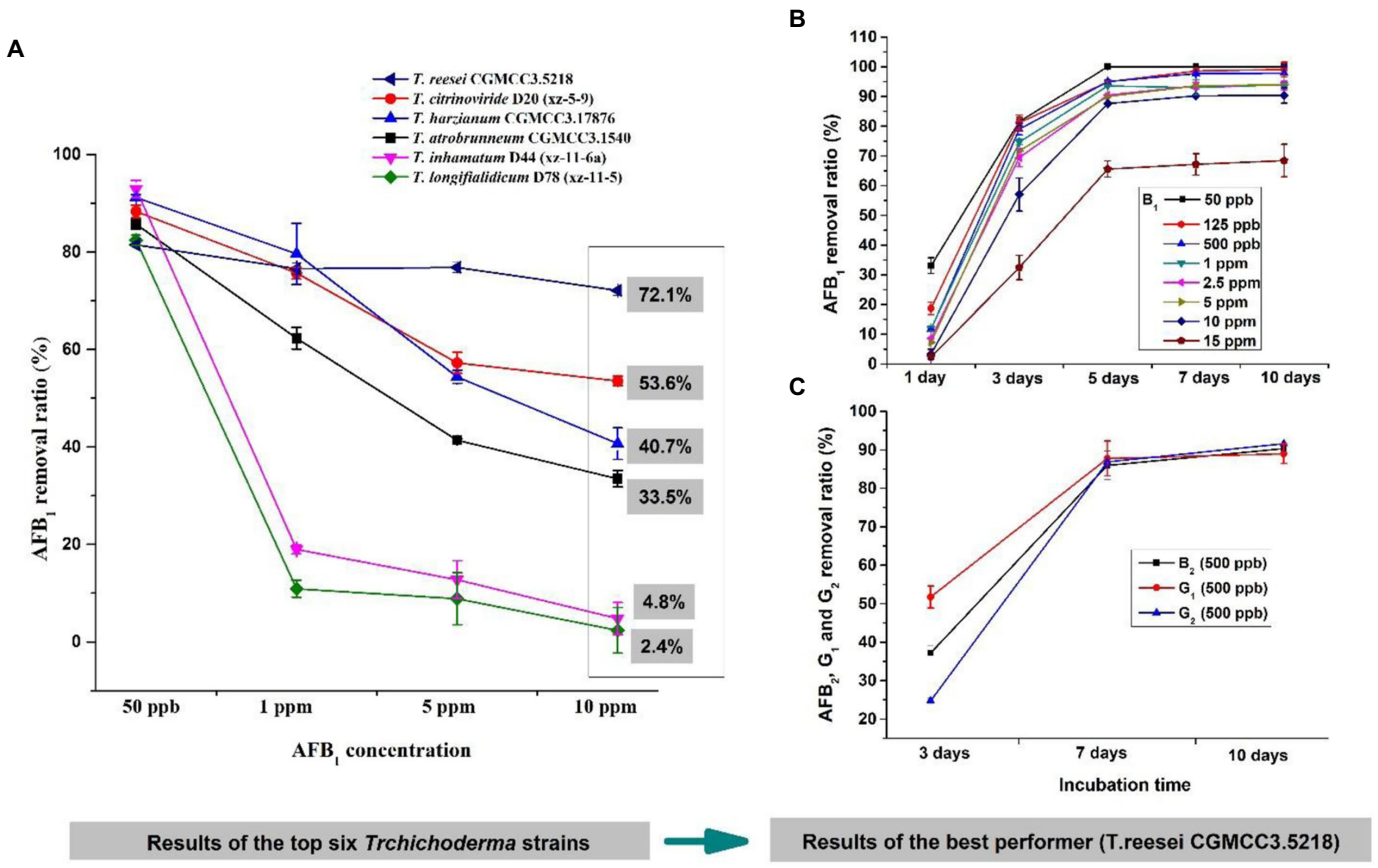
The candidate *Trichoderma* isolates for aflatoxin detoxification. **(A)** Removal ratios of aflatoxin B_1_ (AFB_1_) by *T. reesei* CGMCC3.5218, *T. citrinoviride* D20 (xz-5-9), *T. harzianum* CGMCC3.17876, *T. atrobrunneum* CGMCC3.1540, *T. inhamatum* D44 (xz-11-6a), and *T. longifialidicum* D78 (xz-11-5) cultured in yeast extract peptone dextrose (YPD) liquid medium, after 3 days of incubation at 28°C in the dark. **(B)** Removal ratios of AFB_1_ at concentrations of 50 ng/kg to 15 μg/kg by *T. reesei* CGMCC3.5218 after 1, 3, 5, 7, and 10 days of incubation. **(C)** Removal ratios of 500 ng/kg AFB_2_, AFG_1_, and AFG_2_ by *T. reesei* CGMCC3.5218 cultured in YPD liquid medium, after 3, 7, and 10 days of incubation at 28°C in the dark.

To further determine which isolate was the most promising potential for aflatoxin detoxification, we tested the AFB_1_ removal ratios of the top six isolates. The result displayed that the removal ratio by CGMCC3.5218 was 72.1% at 10 μg/kg, which was significantly higher than those of D20 (xz-5-9), CGMCC3.17876, CGMCC3.1540, D44 (Xz-11-6a), and D78 (xz-11-5) (Figure 3A), suggesting that CGMCC3.5218 had the strongest potential to degrade high doses of AFB_1_. After 5 days of incubation, the removal rates of AFB_1_ at concentrations of 50 ng/kg, 125 ng/kg, 500 ng/kg, 1 μg/kg, 2.5 μg/kg, 5 μg/kg, 10 μg/kg, and 15 μg/kg were 100.0, 94.9, 95.0, 93.6, 90.4, 90.0, 87.6, and 65.6%, respectively (Figure 3B). Additionally, after 10 days of incubation, the removal rates of 500 ng/kg AFB_2_, AFG_1_, and AFG_2_ were higher than 90% (Figure 3C). On the basis of these results, CGMCC3.5218 proved to be extremely effective in removing AFB_1_, AFB_2_, AFG_1_, and AFG_2_. Furthermore, the strain could tolerate extreme conditions of high doses of aflatoxins, with no significant inhibitory effects on its removal capacity even at AFB_1_ concentrations as high as 15 μg/kg. Work by others has demonstrated that various microorganisms and their metabolites have abilities to remove aflatoxins. For example, the cell-free supernatant of microbial consortium TADC7 degraded 95.7% of AFB_1_ at levels of 5 μg/kg (Wang et al., 2017). The percentages of AFB_1_ (0.5 μg/kg) bound by *Saccharomyces cerevisiae* ranged from 81.0 to 99.3% in one study (Gonçalves et al., 2015). *Pseudomonas aeruginosa* could degrade 100 ng/kg AFB_1_ by 82.8% (Sangare et al., 2014). The fungus *Rhizopus oryzae* reduced 100 ng/kg aflatoxin B_1_ by approximately 100% (Hackbart et al., 2014). In this study, however, *T. reesei* CGMCC3.5218 was demonstrated to degrade 10 μg/kg AFB_1_ by approximately 95%. To the best of our knowledge, it was the most capable of removing higher doses of aflatoxins when compared with other strains of bacteria or fungi. The colony of *T. reesei* CGMCC3.5218 grows fast (2–3 cm/day), and the back of the fresh colony is obviously yellow. Additionally, the strain of *T. reesei* CGMCC3.5218 can produce yellow-green conidia after growing for at least 3 weeks. After 2 weeks of growth, the hyphae on the colony surface become hard and dense.

### Optimal culture conditions for the *Trichoderma reesei* CGMCC3.5218 to remove aflatoxins

**Figure 4:**
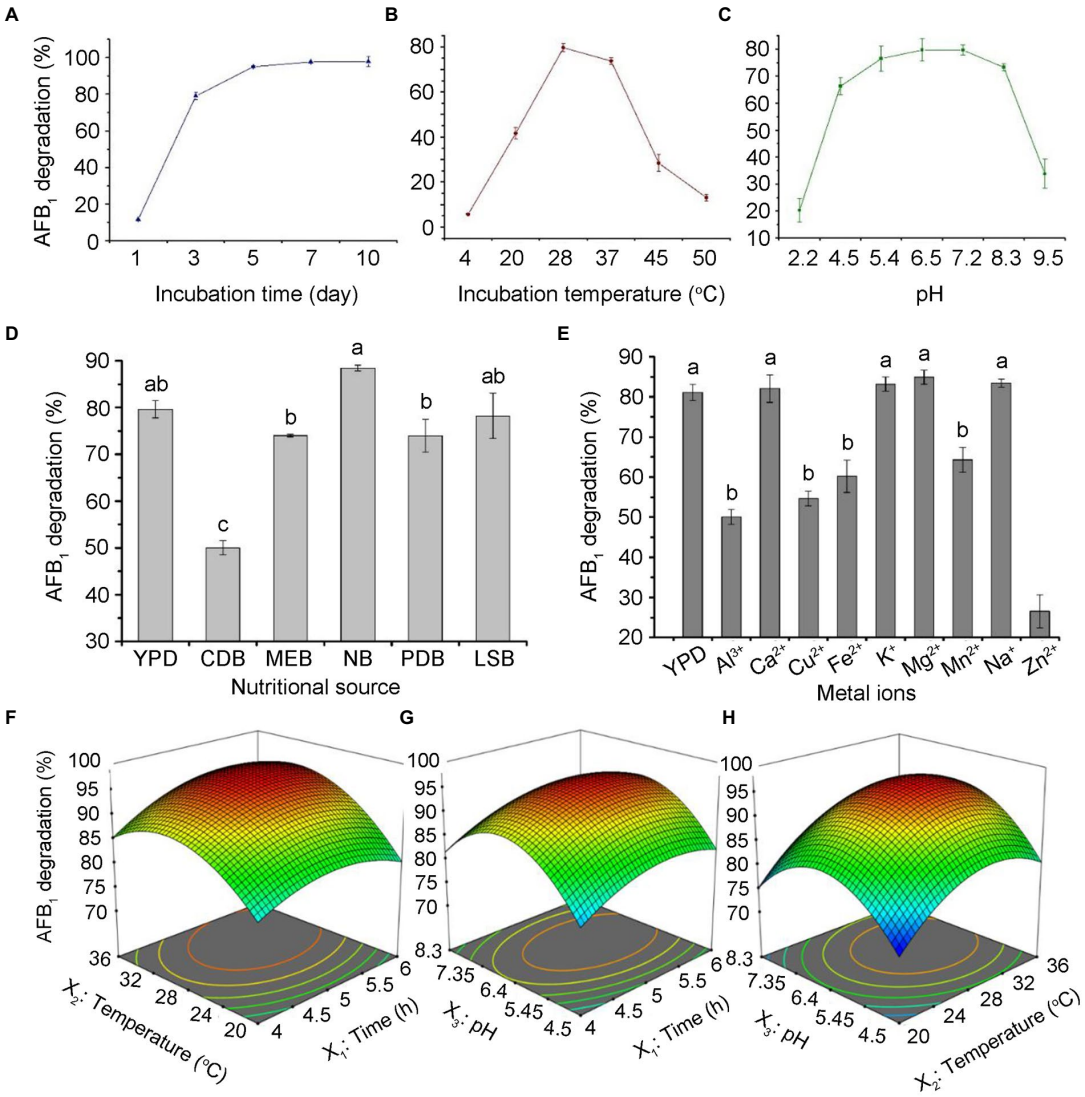
Optimizing culture conditions for CGMCC3.5218 to remove aflatoxins. Effect of **(A)** incubation time, **(B)** culture temperature, **(C)** pH, **(D)** nutritional sources, and **(E)** metal ions on the degradation of 500 ng/kg aflatoxin B_1_ (AFB_1_) by *T. reesei* CGMCC3.5218. Response plots between two parameters for the degradation rate of aflatoxin B_1_: **(F)** Incubation time (X_1_) and culture temperature (X_2_); **(G)** incubation time (X_1_) and medium pH (X_3_); and **(H)** culturing temperature (X_2_) and medium pH (X_3_). The values are the means of three replicates and their standard errors. Bars with different letters are significantly different for *p* < 0.001 (Tukey–Kramer multiple comparison test).

To determine the effects of incubation time, pH, temperature, medium, and metal ions on the AFB_1_ removal ability of *T. reesei* CGMCC3.5218, we tested the AFB_1_ removal ratios under these culture conditions treatment, respectively. The results showed that with the increase of incubation time, the removal rates of AFB_1_ gradually increased. The removal rates of AFB_1_ (500 ng/kg), reached the maximum value at 7 days and then increased almost steadily for up to 10 days (Figure 4A). It has been reported that the biological reduction of AFB_1_ can be due to binding or degradation (Verheecke et al., 2016; Adebo et al., 2017a,b). However, AFB_1_ binding is a rapid process and AFB_1_ can be released back into solutions to some extent with the extension of incubation period (Peltonen et al., 2001; Mwakinyali et al., 2019). Therefore, the removal rates of AFB_1_ by CGMCC3.5218 in different incubation conditions were shown in Figures 4B–E, indicating temperature and pH had significant effects on AFB_1_ removal ability. Particularly, NB medium resulted in an increase of AFB_1_ degradation, whereas the opposite was true with Zn^2+^.

Meanwhile, to further obtain the optimal efficiency of AFB_1_ degradation, we used Box–Behnken (BB) design and response surface methodology (RSM) to optimize the values and interaction effects of the factors that affected the AFB_1_ removal. The Design-Expert 12.0.3 software was used to perform regression analysis on the data in Supplementary Table S3. The detailed analysis of results of the BB test were shown in Supplementary Table S5, and the regression equation was: *Y* = 95.25 + 2.04×_1_ + 4.51×_2_ + 1.74×_3_ + 1.61X_1_X_2_–0.30X_1_X_3_ + 0.75X_2_X_3_ 3.92×_12_ 6.87×_22_ 9.78×_32_, where *Y* refers to the predicted value (percentage of AFB_1_ degradation), X_1_ refers to the incubation time, X_2_ refers to the incubation temperature, and X_3_ refers to the pH of culturing medium. As shown in Supplementary Table S5, the model *F*-value of 729.80 implied that the model was significant. The lack of fit *F*-value of 0.15 implied that the lack of fit was not significant relative to the pure error. The *R*^2^ value of 0.9989 implied that the model fitted the actual experimental results perfectly. *p*-values less than 0.05 indicate that model terms are significant. In this case, X_1_, X_2_, X_3_, X_1_X_2_, X_2_X_3_, X_12_, X_22_, and X_32_ were significant model terms. The response surface and contour map (Figures 4F–H) intuitively reflected the influence of various factors on the response value. The effect of X_2_ on AFB_1_ degradation was more significant than X_1_ and X_3_, as seen in the curve variation degree and contour density. Taken together, the optimal culture conditions were predicted to be at a pH of 6.7, an incubation temperature of 31.3°C, and an incubation time of 5.1 days, as determined by the software Design-Expert 12.0.3. The AFB_1_ degradation was 96.2% under the optimal culture conditions.

### AFB_1_ removal patterns by the CGMCC3.5218

To determine the AFB_1_ removal patterns, we tested the AFB_1_ removal ability among diverse cell component of *T. reesei* CGMCC3.5218. As shown in Table 1, the AFB_1_ removal ratio by extracellular component (91.8%) was significantly higher than that of either active component (8.9%) or intracellular component (19.5%). Additionally, the results also indicated that very small amounts of AFB_1_ were washed out from the mycelia by PBS (0.8 ± 0.2 ng/kg), methanol (0.6 ± 0.1 ng/kg), and acetonitrile (1.6 ± 0.1 ng/kg) compared with the initial concentration (control) of 490.7 ng/kg (Table 1). However, the AFB_1_ absorbed by cell walls could be released back into the solution by elution with polar solvents (Peltonen et al., 2001; Haidukowski et al., 2019). These findings indicated that the AFB_1_ removal was performed by extracellular components rather than intracellular components or adsorption on the cell components. This was in agreement with another report, in which *T. reesei* strain QM9414 reduced approximately 100% of AFB_1_ within 96 h through metabolic degradation by extracellular metabolites (Hackbart et al., 2014). Other studies also have reported similar results. The strain *Lactobacillus plantarum* FJS003 degraded 75.2% AFB_1_ through the fermentation supernatant, which was higher than that (11.4%) degraded by the cells (Zhu et al., 2021). Aflatoxin B_1_ degradation was 58.2% after being treated with *Aspergillus niger* ND-1 culture supernatant for 24 h (Zhang et al., 2014). The cell-free supernatant of isolate *Bacillus subtilis* JSW-1 degraded aflatoxin B_1_ effectively, whereas the viable cells and intracellular extracts were far less effective (Xia et al., 2017). It was further confirmed that the degradation mechanism by culture supernatant was enzymatic (Oluwafemi et al., 2017; Wang et al., 2017).

**Table 1:** Difference of AFB_1_ removal ability among diverse cell components.

Component	AFB_1_ residue ± SD (ng/kg)		Removal ratio (%)
Control	477.5 ± 2.9	*A	
Active component	435.2 ± 3.2	B	8.9
Intracellular component	384.5 ± 4.4	C	19.5
Extracellular component	39.0 ± 5.9	D	91.8
**Item**	**AFB_1_ ± SD (ng/kg)**		**Adsorbed by mycelia (%)**
Control	490.7	^*^A	
PBS	0.8 ± 0.2	B	0.2
Methanol	0.6 ± 0.1	B	0.1
Acetonitrile	1.6 ± 0.1	B	0.3

* AFB_1_ residues followed by different capital letters are statistically different according to Tukey–Kramer Multiple Comparison test (*p* < 0.001).

### Dynamics of AFB_1_ degradation by the CGMCC3.5218 culture supernatant

**Figure 5:**
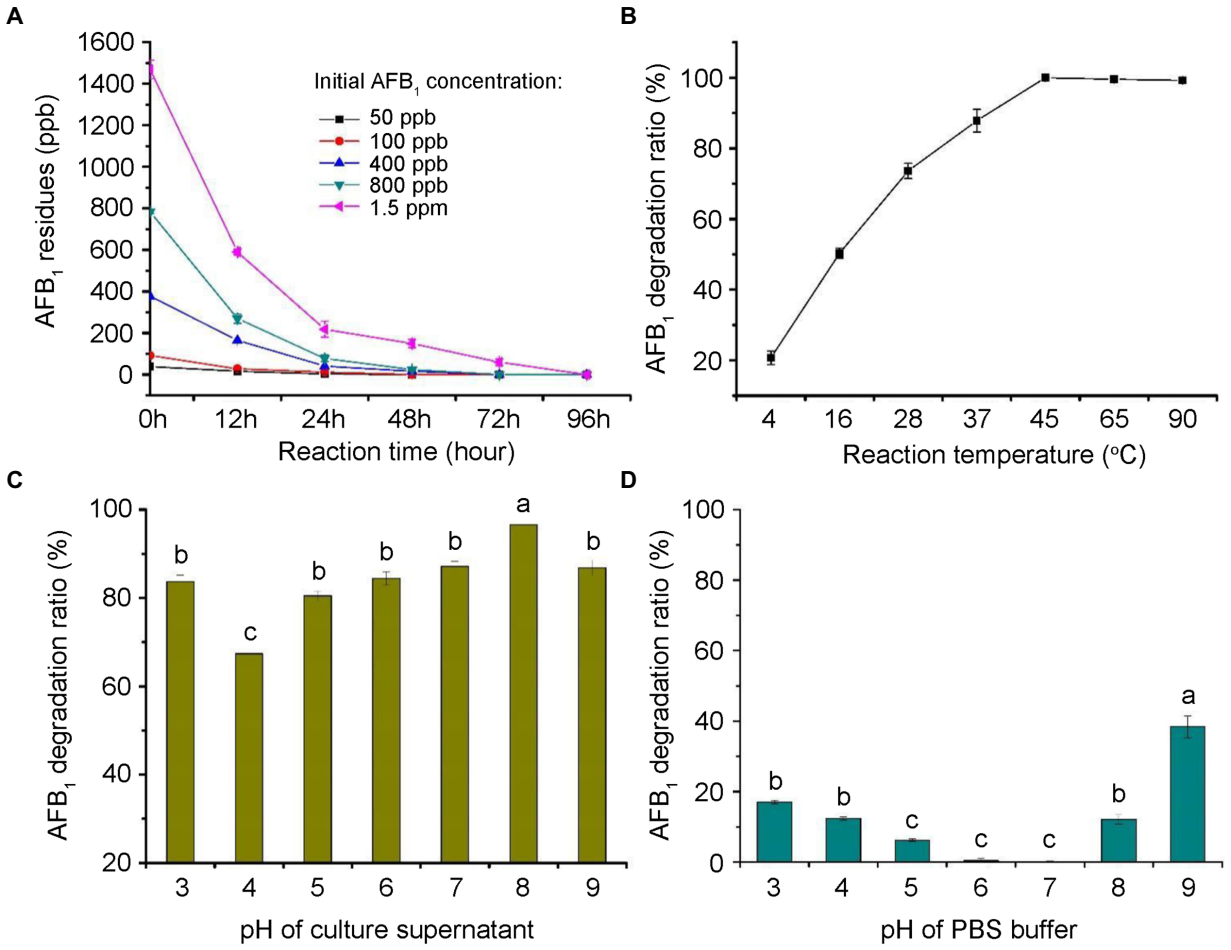
Degradation dynamics of aflatoxin B_1_ (AFB_1_) by the culture supernatant of *T. reesei* CGMCC3.5218. **(A)** Degradation of 50, 100, 400, 800 ng/kg, and 1.5 μg/kg AFB_1_ at various reaction time (0, 12, 24, 48, 72, and 96 h). **(B)** Degradation of 500 ng/kg AFB_1_ at various temperatures of 4, 16, 28, 37, 45, 65, and 95°C. **(C,D)** Degradation of AFB_1_ at a range of pH (3.0–9.0) in culture supernatant and PBS buffer. Bars with different letters are significantly different for p < 0.001 (Tukey–Kramer multiple comparison test). The values are the means of three replicates and their standard errors.

As shown in Figure 5A, most of the degradation by the culture supernatant occurred within 24 h, with calculated AFB_1_ degradation rates of 85.4–100%. As shown in Figure 5B, the AFB_1_ degradation by the culture supernatant was temperature-sensitive within the range of 4°C–45°C. The maximum degradation occurred at 45°C (100%). Interestingly, the degradation percentages of AFB_1_ were approximately the same in the range of 45°C–95°C. This is consistent with the study, in which the degradation activity of the supernatant of microbial consortium TADC7 exhibited thermal stability in range of 45°C–90°C (Wang et al., 2017). These results imply that the enzymes or proteins involved in the AFB_1_ degradation are thermostable. As shown in Figure 5C, the data indicated that CGMCC3.5218 culture supernatant was able to efficiently degrade AFB_1_ in a wide range of pH (3.0–9.0), which would increase its practical use in the future. The AFB_1_ degradation was still high even under strongly acidic and alkali conditions of pH 3.0 and 9.0, respectively. This result imply that pH may contribute to the degradation of AFB_1_. To investigate this possibility, the effects of different pH values of PBS on the degradation of AFB_1_ were studied. As shown in Figure 5D, the degradation rates were 17.0, 12.4, 12.1, and 38.4% at pH 3.0, 4.0, 8.0, and 9.0, respectively. This indicated that AFB_1_ could be destroyed to a certain extent when it was exposed to strongly acidic or strongly alkaline conditions.

### Enzymatic effects of the supernatant on AFB_1_ degradation

**Figure 6:**
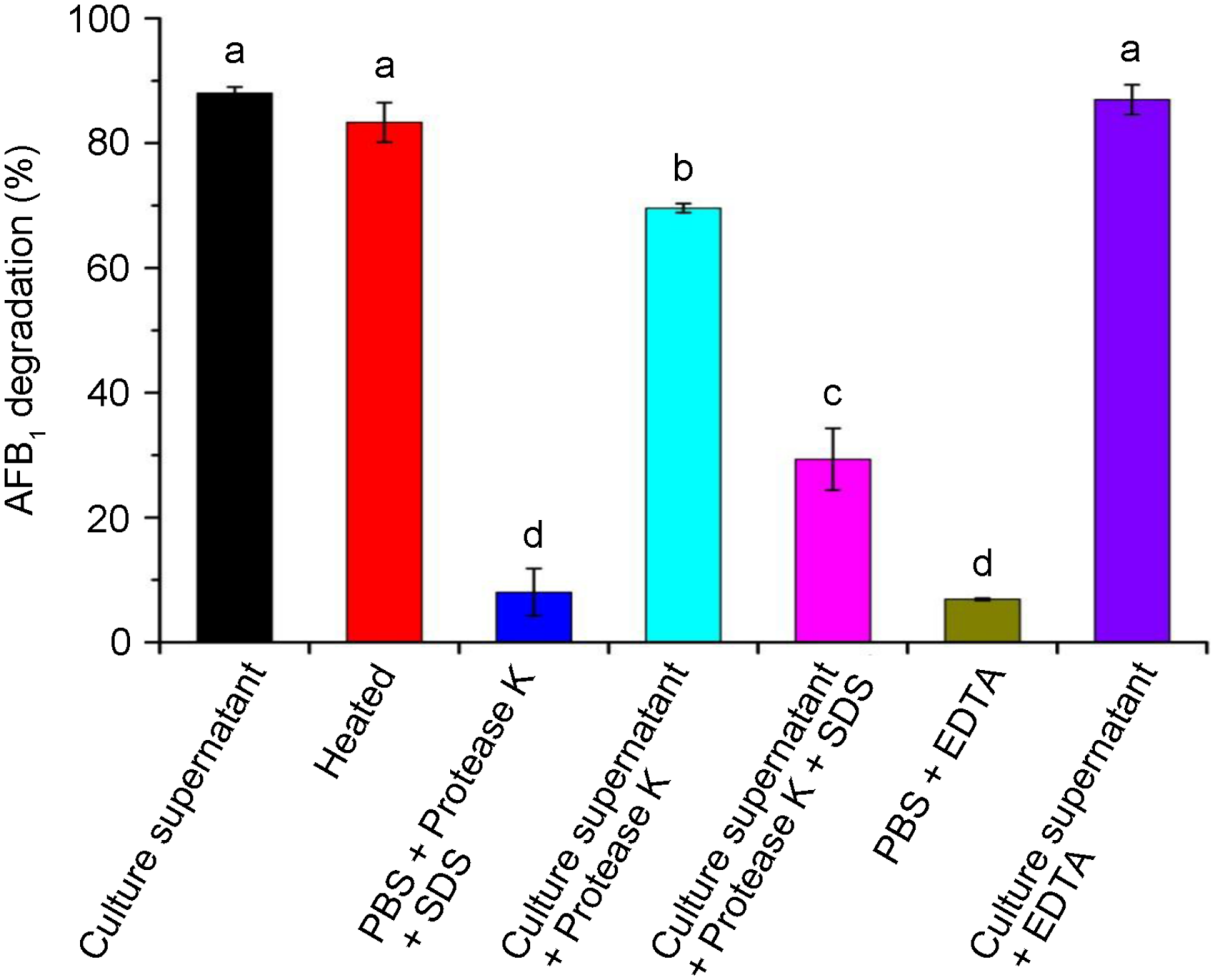
Effects of heat, protease K, protease K plus sodium dodecyl sulfonate (SDS), and Ethylene diamine tetraacetic acid (EDTA) on the aflatoxin B_1_ (AFB_1_) degradation activity of the culture supernatant of *T. reesei* CGMCC3.5218. Bars with different letters are significantly different for *p* < 0.001 (Tukey–Kramer multiple comparison test). The values are the means of three replicates and their standard errors.

To confirm the actively extracellular components were proteins or enzymes, we investigated the enzymatic effects in CGMCC3.5218 culture supernatant. As shown in Figure 6, about 88.0% of AFB_1_ were degraded by the active culture supernatant after incubation for 24 h at 37°C. After a treatment with heat at 121°C for 30 min, AFB_1_ degradation was slightly reduced to 83.3%, suggesting that the enzymes or proteins responsible for AFB_1_ degradation were extremely thermotolerant. EDTA, as a metal chelating agent, did not appear to destroy the AFB_1_ degradation activity (87.0%). However, AFB_1_ degradation declined significantly to 69.6% after treatment with proteinase K, and declined to 29.4% after treatment with proteinase K plus SDS, indicating that enzymes or proteins played a key role in AFB_1_ degradation. Some studies have reported that *T. reesei* has a remarkable ability to secrete proteins or enzymes extracellularly (Sirkka and Merja, 1995; Wang et al., 2015a; Liu et al., 2016). To produce thermotolerant enzymes, a thermotolerant laccase gene from *Pycnoporus sanguineus* and a thermostable β-glucosidase gene from the fungus *Periconia* sp. were successfully cloned and inserted into the chromosomes of *T. reesei* (Dashtban and Qin, 2012; Zhao et al., 2018). However, to the best of our knowledge, this is the first report that *T. reesei* naturally secrete thermotolerant enzymes or proteins involved in contaminant degradation.

### Toxicity analysis of AFB_1_-degraded products

**Figure 7:**
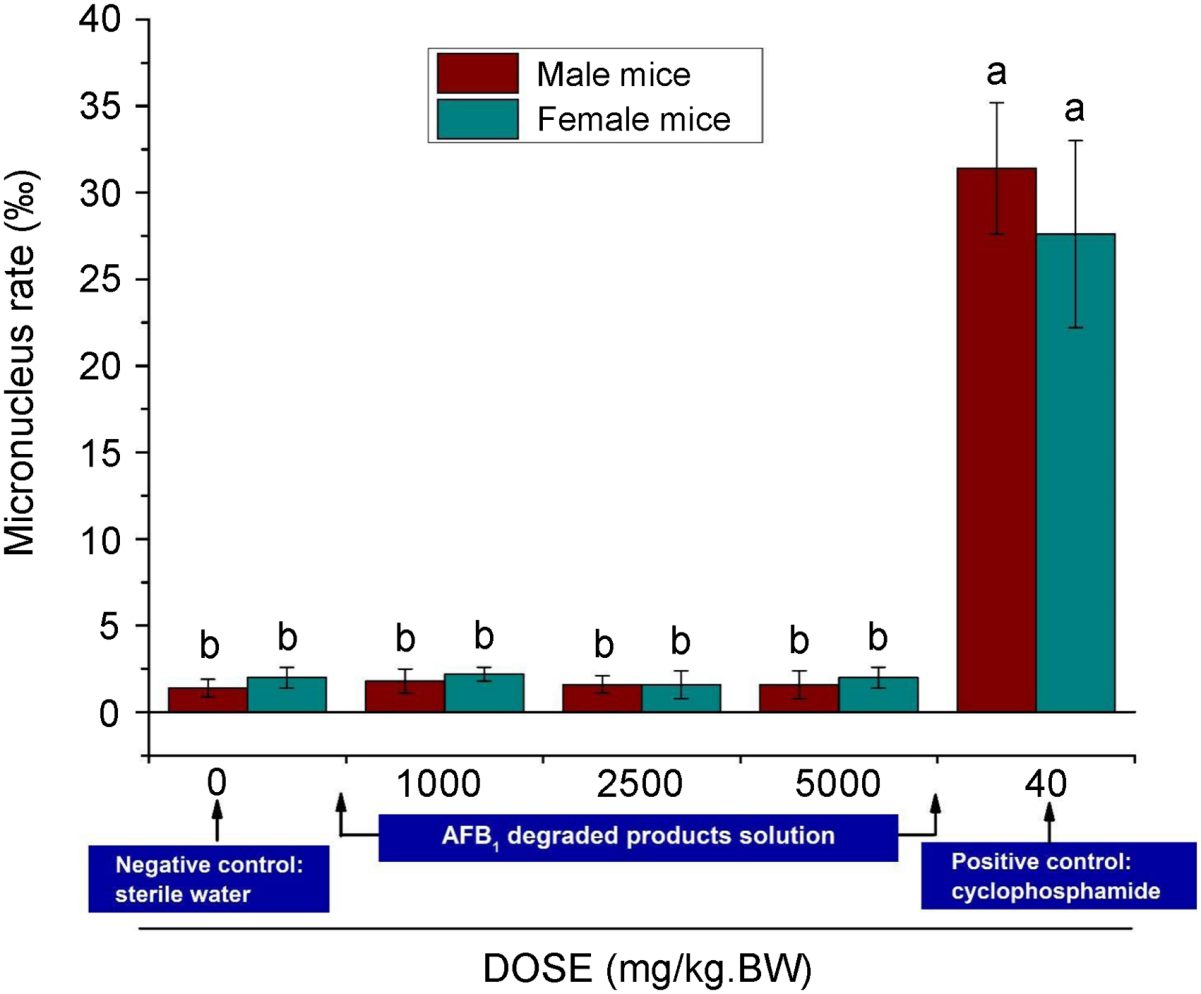
Results of micronucleus test of polychromatic erythrocytes in the bone marrow of male and female mice. Bars with different letters are significantly different for *p* < 0.001 (Tukey–Kramer multiple comparison test).

With regard to aflatoxin degradation, it is conceivable a change in molecular structure may occur, making aflatoxins undetectable, while the toxicity is not necessarily reduced, or is even increased (Detroy and Hesseltine, 1970; Gross-Steinmeyer and Eaton, 2012; Huang et al., 2017). As the aflatoxin degradation potential is not always linked to the reduction of toxicity, it is crucial to determine the toxicity of the degradation products. As shown in Figure 7, the results showed that the micronucleus assay in experimental mouse bone marrow erythrocytes showed no significant differences (*p* > 0.05) from the negative control (sterile water), although there was a significant difference (*p* < 0.01) between the assay and the positive control group (cyclophosphamide), for which the rates of micronucleus were 31.4% for males and 27.6% for females. This indicated that the tested substance in the experimental group did not increase the rate of micronucleus in mouse bone marrow erythrocytes. The acute oral toxicity tests showed that no mice died (Supplementary Table S6). Additionally, no obvious organ lesions were found by macroscopic or histopathological examination on the 14th day after intraperitoneal injection (Supplementary Table S7). The results of skin irritation tests showed that no erythema or edema formed in experimental mice (Supplementary Table S8). All of these results indicated that CGMCC3.5218 can transform the AFB_1_ into nontoxic molecules.

### Aflatoxin degradation by CGMCC3.5218 in peanut cake, maize, and completed feed

To test the application of *T. reesei* CGMCC3.5218 and whether there was a loss of viability and detoxification activity in an actual system, we evaluated degradation of AFB_1_, AFB_2_, AFG_1_, and AFG_2_ in the media containing contaminated peanut cake, maize, and completed feed. As shown in Table 2, when the strain was grown in the media for 7 days, the degradation rates were 85.0–92.1%. Aflatoxin concentrations were significantly reduced compared to the relevant controls. The treatment with CGMCC3.5218 reduced the extremely high aflatoxin content of naturally contaminated maize from 2323.5 μg/kg down to 267.3 μg/kg. These results indicate that CGMCC3.5218 has a noteworthy potential for practical applications for aflatoxin degradation in actual samples.

**Table 2:** Aflatoxins degradation by *T. reesei* CGMCC3.5218 grown on sample substrate.

Sample	Control (B_1_ + B_2_ + G_1_ + G_2_) (μg/kg)	After 1 day		After 3 days		After 7 days	
		Conc. ± SD (μg/kg)	Degradation (%)	Conc. ± SD (μg/kg)	Degradation (%)	Conc. ± SD (μg/kg)	Degradation (%)
^*^A-Peanut	237.2 ± 14.5	182.0 ± 10.4	23.3	93.2 ± 11.6	60.7	25.6 ± 7.2	89.2
A-Maize	184.5 ± 17.9	153.1 ± 13.8	17.0	58.9 ± 13.7	68.1	14.5 ± 4.4	92.1
A-Feed	108.2 ± 11.3	79.5 ± 9.0	26.5	33.9 ± 7.7	68.7	16.2 ± 9.6	85.0
^#^N-Maize	2323.5 ± 92.1	1975.5 ± 46.2	15.0	945.1 ± 70.6	59.3	267.3 ± 49.3	88.5

^*^A-Peanut means artificially contaminated peanut. ^#^N-Maize means naturally contaminated maize.

## Conclusion

*Trichoderma* species are widely found in soils worldwide. Members of this genus have a long history of use as biofertilizer and are generally recognized as safe fungi, whereas very few reports on their use in the biodegradation of small molecular contaminants have been published so far. In this study, AFB_1_ degrading capabilities of 65 *Trichoderma* isolates belonging to 23 different species were investigated. *T. reesei* CGMCC3.5218, which possessed superior efficiency and potential for application in aflatoxin degradation, was selected for further study. The culture conditions for CGMCC3.5218 to degrade AFB_1_ were optimized; the possible removal mechanisms and the enzymatic effects were explored, which indicated that the key AFB_1_ degradation activity of the strain were attributed to the thermostable enzymes or proteins in CGMCC3.5218 culture supernatant. Finally, we demonstrated that this strain could be efficiently used in peanut cake, maize and animal feeds without posing any toxicity risk. When considered together, it can be concluded that the strain *T. reesei* CGMCC3.5218, which has proven to possess strong aflatoxin detoxification ability, has a great potential to be used as a safe and effective biological detoxifier of AFB_1_ in agro-products, foods and feeds. Further work on the purification and characterization of the thermostable enzymes from *T. reesei* CGMCC3.5218 supernatant, as well as the identification of the structures of degradation products or degradation pathways and the possible simultaneous degradation of coexisting contaminants, are required to harness the full potential of *T. reesei* CGMCC3.5218.

## Data availability statement

The datasets presented in this article are not readily available because unrestricted. Requests to access the datasets should be directed to XY xiaofengl19870207@163.com.

## Author contributions

XY, XR, QZ, and PL conceived and designed the experiments. XY, XR, JF, NW, MH, AL, and QZ performed the experiments. XY, XR, CA, and PL analyzed the data. XY, XR, QZ, and PL wrote the paper. All authors contributed to the article and approved the submitted version.

## Funding

This work was supported by the key project of National Natural Sciences Foundation of China (Grant No. 32030085), the Agricultural scientific and technological innovation project of Shandong Academy of Agricultural Sciences (Grant No. 37000021P11000111320E), the National Natural Science Foundation of China (Grant Nos. 31860473 and 32161143034), the Major project of Hubei Hongshan Laboratory (Grant No. 2021hszd015), and the European Union’s Horizon 2020 Research and innovation program under Grant Agreement No. 678781 (MycoKey). The funders had no role in study design, data collection and analysis, decision to publish, or preparation of the manuscript.

## Conflict of interest

The authors declare that the research was conducted in the absence of any commercial or financial relationships that could be constructed as a potential conflict of interest.

## Publisher’s note

All claims expressed in this article are solely those of the authors and do not necessarily represent those of their affiliated organizations, or those of the publisher, the editors and the reviewers. Any product that may be evaluated in this article, or claim that may be made by its manufacturer, is not guaranteed or endorsed by the publisher.
