# Supplementary material for: Characterization and mechanism of aflatoxin degradation by a novel strain of Trichoderma reesei CGMCC3.5218
Source: Front Microbiol. 2022 Oct 13;13:1003039. doi: 10.3389/fmicb.2022.1003039 (PMC9611206; doi:10.3389/fmicb.2022.1003039)
Supplement: Supplementary file 1 [file Data_Sheet_1.docx]

**Characterization and mechanism of aflatoxin degradation by a novel strain of *Trichoderma reesei* CGMCC3.5218**

Xiaofeng Yue^1,5†^, Xianfeng Ren^2†^, Jiayun Fu^1,5^, Na Wei^4^, Claudio Altomare^3*^, Miriam Haidukowski^3^, Antonio F. Logrieco^3^, Qi Zhang^1,5,6*^, Peiwu Li^1,5*^

**Affiliations:**

^1^ Oil Crops Research Institute of the Chinese Academy of Agricultural Sciences, Wuhan, 430062, PR China

^2^ Institute of Quality Standard and Testing Technology for Agro-products, Shandong Academy of Agricultural Sciences, Jinan, 250100, PR China

^3^ Institute of Sciences of Food Production, National Research Council, Bari, 70126, Italy

^4^ Institutions of Agricultural Product Quality Standrad and Testing Research, Tibet Academy of Agricultural and Animal Husbandry Sciences, Lhasa, 850000, PR China

^5^ Key Laboratory of Biology and Genetic Improvement of Oil Crops, Ministry of Agriculture and Rural Affairs, Wuhan, 430062, PR China

^6^ Hubei Hongshan Lab, Wuhan, 430062, PR China

^†^ These authors contributed equally to this work.

^*^ Corresponding authors addressed at:

Institute of Sciences of Food Production, National Research Council, 70126 Bari, Italy; E-mail address: [claudio.altomare@ispa.cnr.it](mailto:claudio.altomare@ispa.cnr.it) (C.A). Tel +39 80 592 9318.

and

Oil Crops Research Institute of the Chinese Academy of Agricultural Sciences, Wuhan, 430062, PR China. E-mail address: zhangqi01@caas.cn (Q. Z.)； peiwuli@oilcrops.cn (P. Li). Tel.: +86 27 86812943; Fax: +86 27 86812862.

Table S1 The geographical origin and screening source of the *Trichoderma* strains, and the AFB_1_ removal ratios by different *Trichoderma* isolates grown in yeast extract peptone dextrose (YPD) medium supplemented with 50 ppb of AFB_1_, after 1, 3 and 7 days of incubation at 28˚C in dark.

| **Species** | **Strains** | **Geographical origin** | **Source** | **After 1 day** | | **After 3 days** | | **After 7 days** | |
| --- | --- | --- | --- | --- | --- | --- | --- | --- | --- |
|  |  |  |  | **Removal ± SD (%)** | | **Removal ±SD**  **(%)** | | **Removal ± SD**  **(%)** | |
| *T. asperellum* | D2 (CYHS02-6) | Tibet, China | Soil | 6.8 ± 2.8 | ^★^A | 32.0 ± 4.6 | A | 39.6 ± 4.2 | A |
|  | D3 (CYHS02-4) | Tibet, China | Soil | 4.1 ± 4.5 | A | 23.7 ± 6.3 | A | 30.1 ± 4.7 | A |
|  | D4 (CYHS02-11) | Tibet, China | Soil | 6.3 ± 4.1 | A | 17.8 ± 9.0 | A | 23.8 ± 4.7 | A |
| *T.* *atrobrunneum* | CGMCC3.154 | Not known | Not known | 14.7 ± 5.7 |  | 87.9 ± 4.8 |  | 100 ± 0.0 |  |
| *T.* *latroviride* | CGMCC3.17878 | Yunnan, China | Rotten wood | 11.9 ± 6.2 | A | 38.7 ± 1.6 | A | 79.1 ± 4.7 | A |
|  | CFA 32 | Not known | Not known | 25.1 ± 5.9 | A | 51.9 ± 7.4 | A | 82.3 ± 4.2 | A |
|  | CFA 50 | USA | Corn kernel | 8.1 ± 5.3 | A | 39.1 ± 2.8 | A | 69.6 ± 5.6 | A |
|  | D8 (xz-3-10) | Tibet, China | Soil | 25.1 ± 5.6 | A | 42.8 ± 2.2 | A | 69.4 ± 6.0 | A |
|  | D9 (xz-3-5) | Tibet, China | Soil | 35.7 ± 9.8 | A | 56.5 ± 2.3 | A | 73.9 ± 2.3 | A |
|  | D14 (28XZYM-5) | Tibet, China | Soil | 21.6 ± 4.9 | A | 63.8 ± 4.4 | A | 77.1 ± 3.3 | A |
| *T.* *citrinoviride* | CGMCC3.779 | China | Not known | 17.2 ± 7.6 | C | 84.4 ± 1.7 | A | 86.2 ± 3.6 | B |
|  | CFA 6 | Austria | Forest soil | 20.2 ± 5.9 | BC | 55.6 ± 6.9 | C | 86.0 ± 1.1 | B |
|  | CFA 54 | Not known | Maize | 16.6 ± 5.9 | C | 59.8 ± 5.1 | C | 84.8 ± 1.1 | B |
|  | D19 (xz-5-7) | Tibet, China | Soil | 44.2 ± 1.8 | A | 80.3 ± 4.5 | AB | 89.2 ± 1.0 | B |
|  | D20 (xz-5-9) | Tibet, China | Soil | 36.6 ± 3.5 | ABC | 88.2 ± 1.3 | A | 100 ± 0.0 | A |
|  | D21 (xz-5-1) | Tibet, China | Soil | 42.7 ± 4.1 | AB | 74.4 ± 0.1 | B | 85.5 ± 3.2 | B |
|  | D22 (xz-5-8a) | Tibet, China | Soil | 34.7 ± 2.9 | ABC | 79.4 ± 5.2 | AB | 83.5 ± 3.5 | B |
| *T. dorotheae* | D24 (xz-2-9) | Tibet, China | Soil | 21.8 ± 9.2 | A | 41.8 ± 3.4 | AB | 75.8 ± 4.4 | A |
|  | D25 (xz-1-10) | Tibet, China | Soil | 25.3 ± 5.2 | A | 69.3 ± 3.2 | A | 81.6 ± 3.2 | A |
|  | D26 (xz-1-4) | Tibet, China | Soil | 27.4 ± 4.0 | A | 23.6 ± 6.3 | B | 79.6 ± 3.9 | A |
| *T.* *erinaceum* | CGMCC5.1548 | Jiangxi, China | Fruit body | 17.7 ± 4.4 | A | 51.0 ± 5.1 | A | 84.8 ± 1.9 | A |
|  | D27 (CYHS03-1) | Tibet, China | Soil | 3.7 ± 2.8 | A | 35.2 ± 6.5 | A | 58.8 ± 3.1 | B |
| *T.* *harzianum* | CGMCC3.1284 | Hebei, China | Soil | 21.8 ± 3.6 | ABC | 68.0 ± 4.3 | C | 84.2 ± 2.4 | B |
|  | CGMCC3.17876 | Yunnan, China | Rotten wood | 11.6 ± 5.5 | BC | 91.2 ± 0.6 | A | 100 ± 0.0 | A |
|  | CFA 8 | Italy | Mushroom substrate | －2.3 ± 4.5 | C | 70.2 ± 1.1 | BC | 89.0 ± 1.8 | AB |
|  | CFA 11 | Italy | Corn kernel | 25.7 ± 4.4 | ABC | 76.3 ± 3.7 | BC | 87.2 ± 1.8 | B |
|  | CFA 37 | Italy | Seedling soil mix | 50.1 ± 5.6 | A | 89.0 ± 1.8 | AB | 93.3 ± 1.1 | AB |
|  | CFA 41 | Borneo, Asia | Soil | 19.0 ± 5.9 | ABC | 65.9 ± 6.4 | C | 89.0 ± 1.8 | AB |
|  | CFA 44 | Italy | Mushroom substrate | 11.1 ± 6.2 | BC | 76.3 ± 3.2 | ABC | 92.1 ± 2.1 | AB |
|  | CFA 51 | USA | Corn kernel | 13.5 ± 2.8 | BC | 70.2 ± 2.8 | BC | 88.4 ± 2.1 | AB |

Continued Table S1:

| **Species** | **Strains** | **Geographical origin** | **Source** | **After 1 day** | | **After 3 days** | | **After 7 days** | |
| --- | --- | --- | --- | --- | --- | --- | --- | --- | --- |
|  |  |  |  | **Removal ± SD (%)** | | **Removal ±SD**  **(%)** | | **Removal ± SD**  **(%)** | |
| *T. harzianum* | CFA 61 | Not known | Not known | 19.0 ± 5.3 | ABC | 17.8 ± 5.7 | D | 60.4 ± 4.4 | C |
|  | D40 (xz-11-14） | Tibet, China | Soil | 33.2 ± 7.3 | AB | 83.2 ± 3.6 | AB | 85.1 ± 1.7 | B |
|  | D41 (xz-11-15) | Tibet, China | Soil | 20.8 ± 6.4 | ABC | 78.8 ± 3.3 | ABC | 89.3 ± 0.6 | AB |
|  | CFA908-S | Italy | Olive | －4.7 ± 3.9 | C | 75.6 ± 2.8 | ABC | 89.6 ± 1.1 | AB |
|  | CFA908-WT | - | UV-mutant of ITEM 908 | 12.9 ± 7.0 | BC | 65.9 ± 2.8 | C | 84.2 ± 1.1 | B |
| *T. hispanicum* | D42 (xz-9-15) | Tibet, China | Soil | 9.4 ± 4.2 | A | 29.3 ± 7.2 | A | 75.0 ± 3.1 | A |
|  | D43 (xz-9-9) | Tibet, China | Soil | 13.8 ± 4.0 | A | 34.3 ± 1.2 | A | 77.0 ± 2.4 | A |
| *T. inhamatum* | CFA 36 | Not known | Not known | 47.0 ± 7.0 | A | 85.4 ± 3.2 | A | 87.2 ± 1.8 | A |
|  | D44 (Xz-11-6a) | Tibet, China | Soil | 34.0 ± 3.7 | A | 92.9 ± 1.8 | A | 100 ± 0.5 | A |
| *T. koningii* | CGMCC3.17875 | Yunnan, China | Rotten wood | 34.6 ± 4.1 | A | 63.7 ± 2.7 | A | 94.8 ± 0.3 | A |
|  | CGMCC3.11471 | Henan, China | Soil | 1.6 ± 2.8 | B | 11.5 ± 3.8 | B | 26.8 ± 1.1 | B |
| *T. koningiopsis* | CGMCC5.1243 | China | Not known | 12.8 ± 4.9 | A | 51.8 ± 6.6 | AB | 92.3 ± 1.5 | A |
|  | CGMCC3.6615 | Yunnan, China | Notoginseng rhizosphere | 14.2 ± 5.5 | A | 25.3 ± 6.0 | B | 92.1 ± 1.4 | A |
|  | D46 (CY06-4) | Tibet, China | Soil | 29.4 ± 2.9 | A | 74.5 ± 5.0 | A | 82.6 ± 4.5 | A |
|  | D49 (CYHS03-3) | Tibet, China | Soil | 7.8 ± 7.0 | A | 43.0 ± 6.5 | AB | 60.0 ± 6.5 | B |
|  | D55 (CYHS02-1) | Tibet, China | Soil | 5.2 ± 3.7 | A | 42.2 ± 6.1 | AB | 47.8 ± 5.1 | B |
| *T. longibrachiatum* | CGMCC3.15738 | Sichuan, China | Soil | 21.1 ± 4.6 | A | 43.9 ± 6.5 | A | 91.9±0.7 | A |
|  | CGMCC3.6607 | Yunnan, China | Notoginseng rhizosphere | 14.8 ± 7.9 | A | 27.7 ± 3.7 | A | 92.0±2.0 | A |
| *T. longifialidicum* | D78 (xz-11-5) | Tibet, China | Soil | 30.7 ± 4.8 |  | 82.4 ± 1.1 |  | 100 ± 0.0 |  |
| *T. neokoningii* | D79 (xz-1-8) | Tibet, China | Soil | 37.3 ± 1.7 |  | 75.9 ± 3.2 |  | 88.2 ± 1.6 |  |
| *T. parceramosum* | CFA46 | Italy | Mushroom substrate | 26.3 ± 7.4 |  | 65.9 ± 2.1 |  | 88.4 ± 5.5 |  |
| *T. polysporum* | CFA60 | Italy | Chestnut soil | －6.5 ± 1.1 |  | 33.6 ± 3.8 |  | 68.9 ± 1.8 |  |
| *T. pseudokoningii* | CGMCC3.18014 | Australia | Wood | 16.7 ± 1.5 |  | 37.6 ± 6.7 |  | 92.8 ± 0.7 |  |
| *T. reesei* | CGMCC3.5218 | China | Rotten wood | 33.1 ± 2.7 | A | 81.5 ± 1.5 | A | 100 ± 0.0 | A |
|  | CGMCC3.3711 | Not known | Not known | 31.6 ± 5.6 | A | 79.5 ± 3.3 | A | 84.6 ± 1.0 | A |
| *T. sinoluteum* | CGMCC3.17528 | Jilin, China | ascospore | －4.6 ± 5.5 |  | 21.5 ± 2.1 |  | 38.8 ± 2.5 |  |
| *T. tomentosum* | CGMCC3.17861 | Yunnan, China | Notoginseng rhizosphere | 2.2 ± 4.2 |  | 15.0 ± 5.8 |  | 18.8 ± 4.8 |  |
| *T. velutinum* | D82 (1930.b) | Tibet, China | Soil | 1.3 ± 5.9 | A | 40.0 ± 4.8 | A | 67.1 ± 1.3 | A |
|  | D83 (1930a) | Tibet, China | Soil | 8.1 ± 5.8 | A | 39.8 ± 6.0 | A | 64.5 ± 4.4 | A |
|  | D85 (1914-5a) | Tibet, China | Soil | 6.0 ± 6.6 | A | 39.8 ± 3.5 | A | 55.1 ± 5.1 | A |

Continued Table S1:

| **Species** | **Strains** | **Geographical origin** | **Source** | **After 1 day** | | **After 3 days** | | **After 7 days** | |
| --- | --- | --- | --- | --- | --- | --- | --- | --- | --- |
|  |  |  |  | **Removal ± SD (%)** | | **Removal ±SD**  **(%)** | | **Removal ± SD**  **(%)** | |
| *T. viride* | CGMCC3.1145 | Shandong, China | Soil | 14.7 ± 6.0 | B | 78.8 ± 3.1 | A | 93.3 ± 0.7 | A |
|  | CFA 62 | Italy | Eggplant leaf | －2.9 ± 2.8 | B | 2.0 ± 5.6 | B | 17.8 ± 4.8 | C |
|  | D87 (1916-9a) | Tibet, China | Soil | 53.7 ± 2.1 | A | 60.9 ± 4.4 | A | 68.5 ± 4.7 | B |
| *T. virilente* | D88 (xz-2-11) | Tibet, China | Soil | 25.0 ± 3.6 | A | 47.7 ± 2.3 | A | 80.7 ± 4.9 | A |
|  | D89 (xz-2-7) | Tibet, China | Soil | 17.2 ± 7.6 | A | 35.8 ± 5.6 | A | 86.2 ± 3.6 | A |

^★^Different capital letters following AFB_1_ removal ratios in the column means significant difference of AFB_1_ removal ability within the species for P < 0.001 (Tukey–Kramer Multiple Comparison Test).

Table S2 Factors and coded value of Box-Behnken design.

| **Term** | **Variable** | **Coded level** | | |
| --- | --- | --- | --- | --- |
|  |  | **-1** | **0** | **1** |
| A | Incubation time (day) | 4 | 5 | 6 |
| B | Temperature (℃) | 20 | 28 | 36 |
| C | pH | 4.5 | 6.4 | 8.3 |

Table S3 Box-Behnken experimental design matrix and response values.

| **Run** | **Incubation time (A)** | **Temperature**  **(B)** | **pH**  **(C)** | **AFB_1_ degradation (Y)** |
| --- | --- | --- | --- | --- |
| 1 | ﹣1 | 0 | ﹣1 | 77.5 |
| 2 | ﹣1 | 0 | 1 | 81.5 |
| 3 | 1 | 0 | ﹣1 | 81.2 |
| 4 | 1 | ﹣1 | 0 | 80.3 |
| 5 | 0 | ﹣1 | 1 | 75.2 |
| 6 | 0 | 1 | 1 | 85.6 |
| 7 | 0 | 0 | 0 | 95.0 |
| 8 | 0 | 0 | 0 | 94.8 |
| 9 | ﹣1 | ﹣1 | 0 | 79.5 |
| 10 | 1 | 0 | 1 | 85.0 |
| 11 | 0 | 0 | 0 | 95.0 |
| 12 | 0 | ﹣1 | ﹣1 | 73.1 |
| 13 | 0 | 1 | ﹣1 | 80.5 |
| 14 | 0 | 0 | 0 | 95.9 |
| 15 | 1 | 1 | 0 | 92.7 |
| 16 | ﹣1 | 1 | 0 | 85.0 |
| 17 | 0 | 0 | 0 | 95.6 |

Table S4 Parameters of building the phylogenetic tree.

| **Option** | **Selection** |
| --- | --- |
| Analysis | Phylogeny reconstruction |
| Statistical Method | Maximum likelihood |
| Test of Phylogeny | Bootstrap method |
| No. of bootstrap replications | 500 |
| Substitutions Type | Nucleotide |
| Model/Method | Kimura 2-parameter Model |
| Gaps/Missing Data Treatment | Partial deletion |
| Site Coverage Cutoff | 95 |

Table S5 Variance analysis of regression equation.

| **Source** | **Sum of squares** | **df** | **Mean squares** | **F-value** | **P-value（Prob>F）** |  |
| --- | --- | --- | --- | --- | --- | --- |
| Model | 965.79 | 9 | 107.31 | 729.80 | < 0.0001 | significant |
| X_1_ | 33.45 | 1 | 33.45 | 227.46 | < 0.0001 |  |
| X_2_ | 162.49 | 1 | 162.49 | 1105.09 | < 0.0001 |  |
| X_3_ | 24.31 | 1 | 24.31 | 165.30 | < 0.0001 |  |
| X_1_X_2_ | 10.35 | 1 | 10.35 | 70.41 | < 0.0001 |  |
| X_1_X_3_ | 0.3701 | 1 | 0.3701 | 2.52 | 0.1567 |  |
| X_2_X_3_ | 2.25 | 1 | 2.25 | 15.31 | 0.0058 |  |
| X_1_^2^ | 64.69 | 1 | 64.69 | 439.92 | < 0.0001 |  |
| X_2_^2^ | 198.79 | 1 | 198.79 | 1351.95 | < 0.0001 |  |
| X_3_^2^ | 402.83 | 1 | 402.83 | 2739.58 | < 0.0001 |  |
| Residual | 1.03 | 7 | 0.1470 |  |  |  |
| Lack of Fit | 0.1034 | 3 | 0.0345 | 0.1488 | 0.9252 | not significant |
| Pure Error | 0.9259 | 4 | 0.2315 |  |  |  |
| Cor Total | 966.82 | 16 |  |  |  |  |
| R^2^=0.9989, R_Adj_^2^=0.9968 | | | | | | |

Table S6 The results of acute oral toxicity test of AFB_1_ degradation products degraded by *T. reesei* CGMCC3.5218.

| **Mice Sex** | **Dose (mg/kg.BW)** | **Mice numbers** | **Beginning weight** | **Final weight** | **Death count** | **MTD**  **(mg/kg.BW)** | **LD50**  **(mg/kg.BW)** |
| --- | --- | --- | --- | --- | --- | --- | --- |
| Female | 5000 | 10 | 19.8±0.3 | 34.4±0.6 | 0 | >5000 | >5000 |
| Male | 5000 | 10 | 20.0±0.4 | 41.0±0.6 | 0 | >5000 | >5000 |

Table S7 Acute intraperitoneal injection pathogenicity test of AFB_1_ degradation products degraded by *T. reesei* CGMCC3.5218.

| **Mice Sex** | **Dose (ml)** | **Mice numbers** | **Weight** | **Death count** | **Anatomy observation** | | |
| --- | --- | --- | --- | --- | --- | --- | --- |
|  |  |  |  |  | **D3** | **D7** | **D14** |
| Female | 0.20 | 20 | 19.4±0.5 | 0 | No lesions | No lesions | No lesions |
| Male | 0.20 | 20 | 19.7±0.6 | 0 |  |  |  |

Table S8 Acute transdermal toxicity test of AFB_1_ degradation products degraded by *T. reesei* CGMCC3.5218.

| **Stimulation time (h)** | | **1** | | | | **24** | | | | **48** | | | |
| --- | --- | --- | --- | --- | --- | --- | --- | --- | --- | --- | --- | --- | --- |
| **Serial number of mice** | | **1** | **2** | **3** | **Stimulation Index** | **1** | **2** | **3** | **Stimulation Index** | **1** | **2** | **3** | **Stimulation Index** |
| Sample | Red spot | 0 | 0 | 0 | 0 | 0 | 0 | 0 | 0 | 0 | 0 | 0 | 0 |
|  | Edema spot | 0 | 0 | 0 |  | 0 | 0 | 0 |  | 0 | 0 | 0 |  |
| Control | Red spot | 0 | 0 | 0 | 0 | 0 | 0 | 0 | 0 | 0 | 0 | 0 | 0 |
|  | Edema spot | 0 | 0 | 0 |  | 0 | 0 | 0 |  | 0 | 0 | 0 |  |
